# Cytoplasmic factories, virus assembly, and DNA replication kinetics collectively constrain the formation of poxvirus recombinants

**DOI:** 10.1371/journal.pone.0228028

**Published:** 2020-01-16

**Authors:** Quinten Kieser, Ryan S. Noyce, Mira Shenouda, Y.-C. James Lin, David H. Evans

**Affiliations:** 1 The Dept. of Medical Microbiology & Immunology, University of Alberta, Edmonton, Alberta, Canada; 2 Li Ka Shing Institute of Virology, University of Alberta, Edmonton, Alberta, Canada; Arizona State University, UNITED STATES

## Abstract

Poxviruses replicate in cytoplasmic structures called factories and each factory begins as a single infecting particle. Sixty-years ago Cairns predicted that this might have effects on vaccinia virus (VACV) recombination because the factories would have to collide and mix their contents to permit recombination. We've since shown that factories collide irregularly and that even then the viroplasm mixes poorly. We’ve also observed that while intragenic recombination occurs frequently early in infection, intergenic recombination is less efficient and happens late in infection. Something inhibits factory fusion and viroplasm mixing but what is unclear. To study this, we’ve used optical and electron microscopy to track factory movement in co-infected cells and correlate these observations with virus development and recombinant formation. While the technical complexity of the experiments limited the number of cells that are amenable to extensive statistical analysis, these studies do show that intergenic recombination coincides with virion assembly and when VACV replication has declined to ≤10% of earlier levels. Along the boundaries between colliding factories, one sees ER membrane remnants and other cell constituents like mitochondria. These collisions don't always cause factory fusion, but when factories do fuse, they still entrain cell constituents like mitochondria and ER-wrapped microtubules. However, these materials wouldn’t seem to pose much of a further barrier to DNA mixing and so it’s likely that the viroplasm also presents an omnipresent impediment to DNA mixing. Late packaging reactions might help to disrupt the viroplasm, but packaging would sequester the DNA just as the replication and recombination machinery goes into decline and further reduce recombinant yields. Many factors thus appear to conspire to limit recombination between co-infecting poxviruses.

## Introduction

Poxviruses pursue a coordinated pattern of development over the hours that it takes to complete a single round of infection. This happens in the cytoplasm and is regulated by the scheduled expression of different genes, genes regulated by promoters that are transcribed at early, intermediate, and/or late times in infection. In cells infected by vaccinia virus (VACV), early mRNAs are associated with ribosomes by ~2 hr post-infection and most intermediate and late transcripts by 4-to-8 hr post-infection [1]. The transition from one state to the next is each accompanied by hallmark events. The first few steps broadly encompass virus entry and loss of the wrapping membrane, early mRNA translation, capsid uncoating, DNA release, and initiation of DNA synthesis. The onset of DNA replication coincides with a switch to transcribing intermediate mRNAs and the encoded transcription and other factors [2] then promote a switch to making late mRNAs and the proteins required for virion assembly. Although many details remain to be elucidated, it is apparent that virus-directed proteolytic processing reactions play an important role in driving this process forward. For example, proteasome inhibitors block replication initiation [3] and subsequent research has shown that Cullin-3 [4] and the VACV BTB/Kelch protein C5 [5] play roles in uncoating and DNA release.

Poxvirus DNA replication progresses within cytoplasmic structures originally called Guarnieri bodies and now commonly called factories [6, 7]. Each factory derives from a single infecting particle and at early stages in infection they are compact DNA-containing structures surrounded by membranes derived from the cell’s rough endoplasmic reticulum (ER) [8]. The factories enlarge with continuing DNA synthesis, and gradually adopt a more irregular appearance as cavities form containing viral mRNA and host translation factors [9]. In the later stages of the infection cycle a complex of late gene products including D13, A14, and A17, plus a collective of viral membrane assembly proteins, act to dismantle the surrounding ER membranes and produce crescent-shaped structures as substrates for the assembly of immature virions (IV) [10]. IV are then processed into mature virion (MV). MV are the most abundant infectious species although a small subset will acquire more membranes from the trans-Golgi network [11] and exit the cell by fusion with the cytoplasmic membrane. The cannibalization of the ER to make the IV/MV membrane, and the encapsidation of virus DNA, likely plays important roles in altering the appearance of the factories late in infection.

Poxvirus DNA replication takes place in these factories; catalyzed by VACV E9 DNA polymerase and accessory factors like I3, a single-strand DNA binding protein [12–15]. Curiously, any DNA transfected into infected cells is also replicated within factories by the same poxvirus proteins [16, 17] where it is repeatedly copied and recombined [18, 19]. The inextricable connection between replication and recombination is explained by the fact that E9 and I3 also catalyze a recombination-repair reaction dependent upon single-strand annealing [20, 21]. This recombination machinery would play a critically important role in repairing double-stranded breaks [22] and broken replication structures (reviewed in [23]) and aiding virus evolution [24, 25]. However, previous research has detected some seemingly contradictory features of these reactions. Although co-transfected DNAs recombine so much that linkage is lost at distances >350 bp [18], the recombinants recovered from virus-by-virus crosses exhibit only ~1 crossover per 8–12 kbp [26]. Moreover, there is a significant difference in the timing of the appearance of recombinant gene products depending upon how the crosses are organized. If the recombining gene elements are co-located on the same virus genome, then an abundance of recombinant proteins are detected and early, but if the DNA’s are encoded on separate genomes, then far fewer recombinants are formed and only late in infection after most DNA synthesis has concluded [27]. Why?

We’ve suggested that factories may be responsible for altering the timing and frequency of recombination, an idea first proposed by John Cairns sixty years ago [6].The DNA from two genetically-distinct co-infecting viruses has to mix to permit recombination and such interactions cannot happen until two or more developing factories collide and then often only late in infection [27]. Even then, fluorescence *in-situ* hybridization has shown that these collisions don’t always produce extensive mixing of different virus DNAs [28]. To gain some insights into what factors might be limiting the mixing and recombination of DNA in poxvirus factories, we’ve used a combination of methods, including correlative light and electron microscopy, to examine the fate of VACV factories and their contents at time points up to when recombination between viruses is observed. Our studies show that poxvirus factories are surrounded by a matrix of cytoplasmic constituents and that these components, like mitochondria and ER, could create an impediment that partially inhibits the mixing of different viroplasma. This, plus the properties of the viroplasm itself, create a mixing barrier that is expected to delay the timing and limit the extent of poxvirus recombination even though the recombination and replication machinery is still active late in the infection cycle.

## Results and discussion

### Infection kinetics and virus morphogenesis

**Fig 1:**
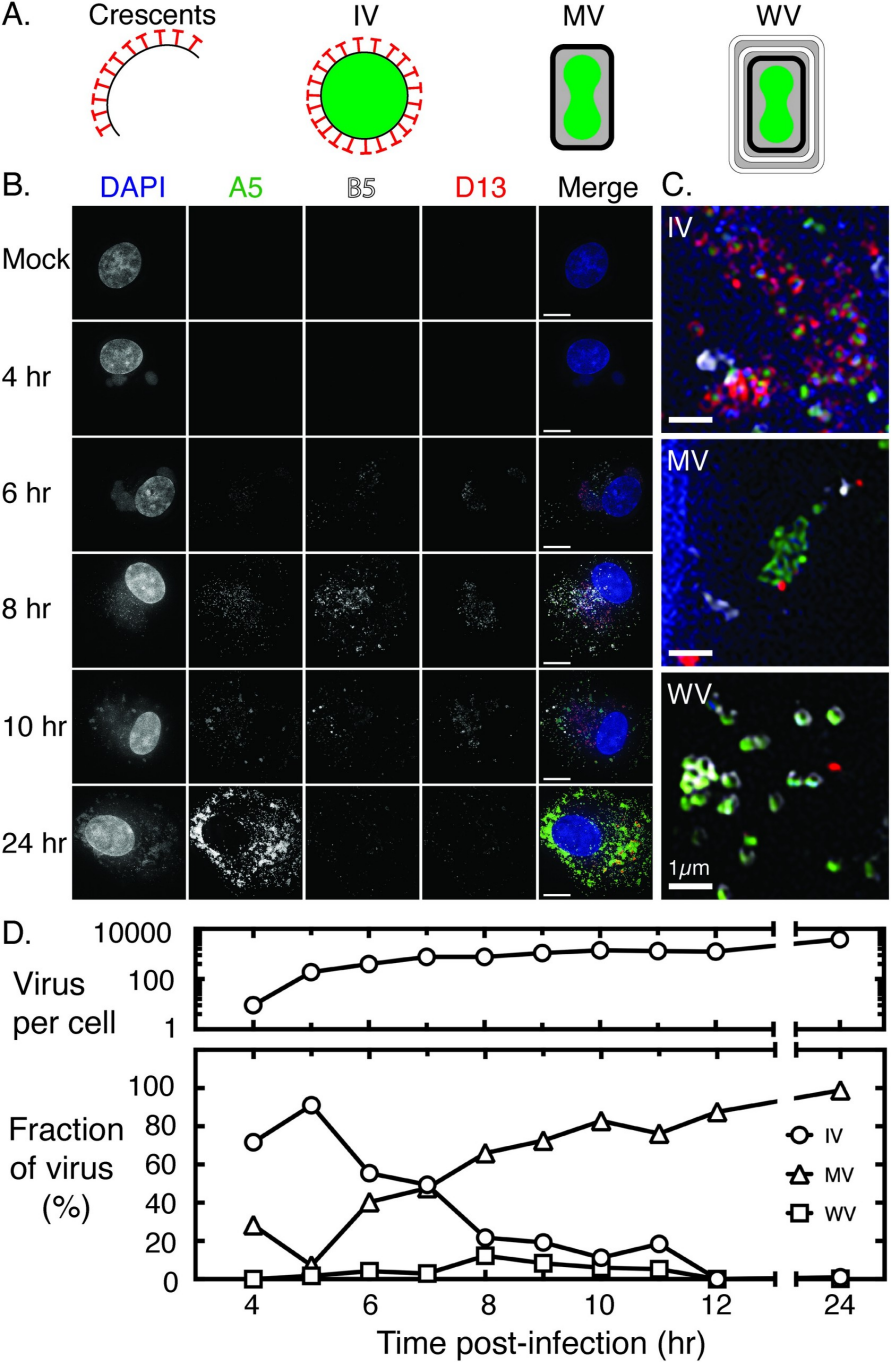
Timing of virus development. BSC40 cells were infected with VACV-A5-YFP at a MOI = 5, fixed at the indicated times, and stained with DAPI and antibodies to D13 and B5. (A) Schematic showing the different developmental forms of VACV. Virus assembly begins with formation of viral crescents that eventually form IV. During maturation, the scaffold that gives shape to both crescents and IV is cleaved and the brick-shaped MV is formed. A fraction of MV migrate towards the trans-Golgi network where they obtain additional membranes (white) and incorporate other unique proteins. (B) Representative images showing the timing of the appearance of each viral form. The YFP-A5 tag detects all three types of virus (MV, IV, and WV), which can be further differentiated using antibodies that detect the D13 scaffolding protein (IV) and the outer envelope protein B5 (WV). Bar = 10μm. (C) An inset of selected regions imaged at 10 hr post-infection. The ring structures formed by D13 (red) and B5 (white) are seen associated with the A5 cores (green). MV tend to aggregate in clusters at later times in infection. Bar = 1μm. (D) Distribution of virus forms at different times post-infection and total numbers of virus particles analyzed. The figure shows data consolidated from 3 independent experiments and reports a number-weighted average of the distribution of morphogenic forms using a total of 10 cells per time point. We measured an average of 1100 virus/cell (range 9–3900 at 4 and 24 hr, respectively).

We’ve previously used fluorescent reporter genes driven from early-late promoters to show that recombinant gene products can be first detected in co-infected cells about 6 hr post-infection and after the onset of late gene expression [27]. Poxvirus late gene products play a key role in virion morphogenesis (reviewed in [29–31]) and so to examine what else was happening around these time points, we tracked the association of three virus proteins (D13, A5 and B5) with nascent virions using super-resolution structured illumination fluorescence microscopy. A5 is incorporated into the virus core early in morphogenesis and is present in all forms of virus. D13 serves as a scaffolding protein and associates with both viral crescents and immature virions. The D13 scaffold is lost as immature virions mature into MV and thus serves as a marker of the early steps in assembly and IV. Finally, B5 is an envelope protein that serves as a marker for another intracellular virus, a form bearing two additional membranes called wrapped virus (WV). These steps in assembly are summarized in Fig 1A.

By simultaneously labelling and imaging the structures associated with these proteins we could differentiate IV (A5+D13), MV (A5 alone), and WV (A5+B5) in infected cells (Fig 1B and 1C). Although the structures begin to approach the limits of resolution of OMX microscope technology (~120 nm), D13 and B5 still appeared to form the ring-shaped assemblies that one would expect considering their roles as scaffold and envelope proteins. We could also measure the proportion of each form relative to the total number of A5-bearing particles in fixed cells imaged at different times after infection (Fig 1D). A few MV (<10) were still detected at the first (4 hr) time point in some cells, but this proportion of virus had dropped significantly an hour later and presumably represented the inoculum. As was expected, IV were the first new form to appear at 4 to 5 hr post-infection and were seen only in DAPI-stained regions of factories (Fig 1B). Thereafter the proportion of IV declined steadily with a half-life of ~1.6 hr. New MV appeared in significant quantities shortly thereafter, although these were mostly found outside of factories, where they later formed large A5-tagged aggregates (Fig 1C). WV also began to appear at about the same time as MV, collectively with a doubling time of ~1.5 hr, but the proportion of WV never exceeded the 10% of virus seen at 8 hr post-infection. These WV were localized toward the cell periphery, near the plasma membrane. By 24 hr post-infection MV comprised >98% virus and cells often contained over 3500 particles per cell. These observations showed that virus assembly reactions are well in progress at the times when recombinant genes are first being detected.

### Persistence of DNA replication

**Fig 2:**
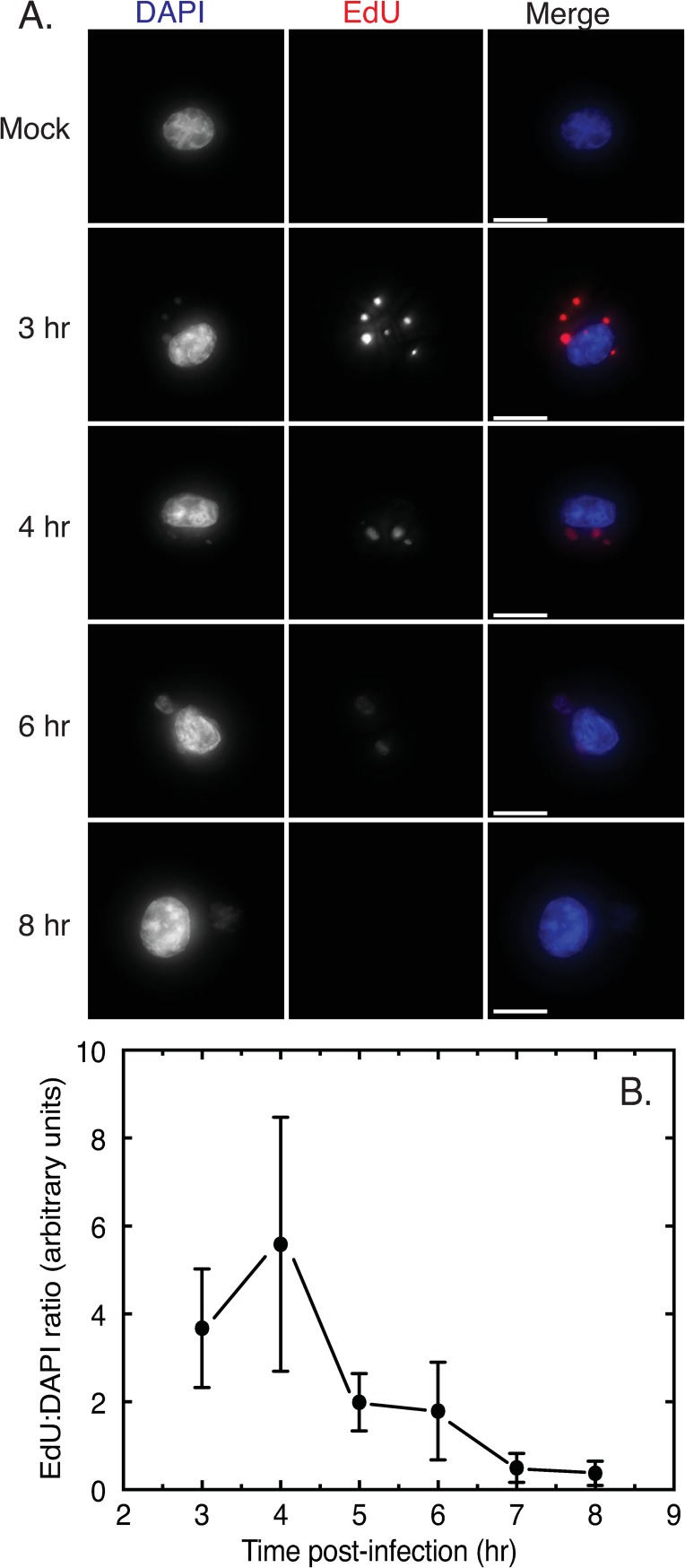
DNA replication through the VACV life cycle. BSC40 cells were infected with VACV strain WR at a MOI = 5 and pulsed with EdU for 15 min prior to fixation at the indicated time points. The EdU was coupled to AlexaFluor 647 dye and the bulk DNA labelled with DAPI. (A) Fluorescence micrographs showing the sites of EdU incorporation throughout the viral life cycle. Nascent factories can be labelled brightly with EdU early in infection, the rate of incorporation declines with time as the factories mature and adopt a more diffuse appearance. Images represent a projection of all z-stacks. Bar = 10μm. (B) Quantification of EdU incorporation. The fluorescence from the AlexaFluor-tagged EdU was normalized relative to the amount of DNA detected using DAPI fluorescence. The figure shows data acquired from three replicate experiments, averaging all signals acquired from 13–19 cells per time point. The error bars show standard deviations.

VACV recombination is catalyzed by the E9 DNA polymerase [21], but this is an early gene and most DNA synthesis is detected early in infection. What’s unclear is whether the DNA replication machinery continues to join, replicate and repair recombinant molecules through the later stages of infection when recombinant gene products are detected. To examine this question, we used a combination of 5-ethynyl-2’deoxyuridine (EdU) pulse-labelling and “click” chemistry to tag sites of DNA replication at different times in the VACV replication cycle (Fig 2). Using cells pulse-labelled for 15 min and then fixed and imaged, we saw that the greatest amounts of EdU were incorporated at early time points (3–4 hr post-infection) and that this label was localized within virus factories and (sometimes) the cell nucleus (Fig 2A). To illustrate this point in a more quantitative manner we measured the ratio of the EdU signal to the DAPI signal intensity (Fig 2B). The rate of EdU incorporation peaked between 3–4 hr post-infection and declined thereafter with a half-time for the decay rate of somewhat less than 2 hr. Little EdU incorporation was detected at the latest time point (8 hr post-infection) although even then some ongoing DNA synthesis could still be detected in the factories at 5–10% of the levels detected in the 3–4 hr period. These kinetics parallel, but lag 1–2 hr behind the kinetics of E9L gene expression [32].

These studies exhibited a lot of cell-to-cell experimental variation (Fig 2B) so we also investigated whether this late DNA synthesis could also be seen at sites and in cells where intergenomic recombination was also detected. To do this, we used live-cell microscopy and two different fluorescently-tagged molecules to track the development of virus factories and to detect the formation of recombinant genomes [27]. Virus DNA replication was monitored using a cell-encoded and DNA binding form of enhanced green fluorescent protein (GFP-cro). Recombination was detected using two different co-infecting VACV, each encoding a partially-duplicated fragment of a DNA-binding form of mCherry fluorescent protein (mCherry-cro). The reconstruction of the mCherry gene by recombination permits expression of a full-length fluorescent protein from an early-late promoter [27]. The DNA binding tag serves to concentrate the recombinant protein in a region near the site(s) of recombination.

**Fig 3:**
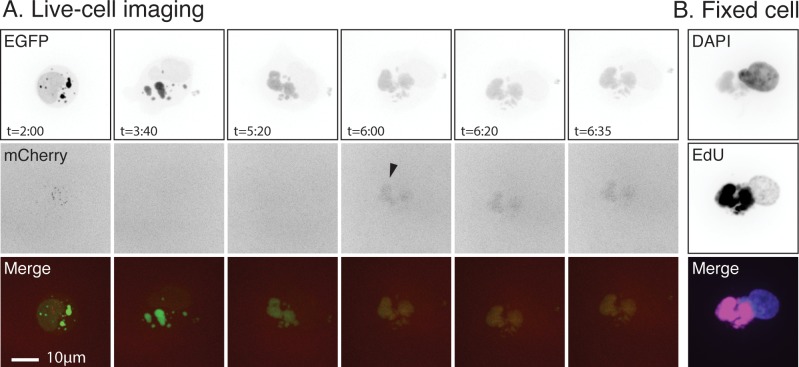
DNA replication associated with inter-viral recombination. BSC40-eGFP-cro cells were infected with VACV-pE/L-mCherry(t) and VACV-pmCherry-cro at a MOI = 2.5 for each virus. The cells were pulse-labelled for 35 min with EdU starting at 6 hr post-infection and fixed a few minutes after the last time point. The EdU was coupled to AlexaFluor 647 dye and the bulk DNA labelled with DAPI. These experiments were completed a total of three times and this figure highlights the results of a single experiment. (A) Still images acquired during the live-cell portion of the experiment. Traces of the recombinant mCherry-cro reporter protein were first seen at 5:20 hr post-infection, but only became obviously visible at 6 hr (arrow). (B) The cell that was tracked during the live-cell portion of the experiment was reimaged after processing to detect any incorporated EdU. Both EdU and recombinant mCherry-cro molecules seem to be distributed throughout the factories. Bar = 10 μm.

BSC40-GFP-cro cells were infected with the two VACV at MOI = 5 and the progress of the infection monitored using live-cell imaging. During the earlier stages of the infection many factories were seen to collide and mix (Fig 3A and S1 Video). At 6 hr post-infection EdU was added to the specimen and the infection then allowed to proceed for another 35 min before being stopped, fixed, and processed to detect any newly incorporated EdU. In this particular specimen, the first signs of recombination between two co-infecting particles (i.e. mCherry fluorescence) were detected at 5:20 hr post-infection (S1 Video) although it isn’t clearly visible in the still images until 6:00 hr (Fig 3A). After processing the sample to label the DNA and the sites of EdU incorporation, the grid marks on the dishes were used to relocate the cells that had been imaged during the live-cell portion of the experiment (Fig 3B). The factories that had merged and produced recombinant mCherry had also incorporated EdU, showing that the replication machinery was still present and active in factories during and even after when recombinant formation was first observed.

### Optical and electron imaging of the ER boundaries

**Fig 4:**
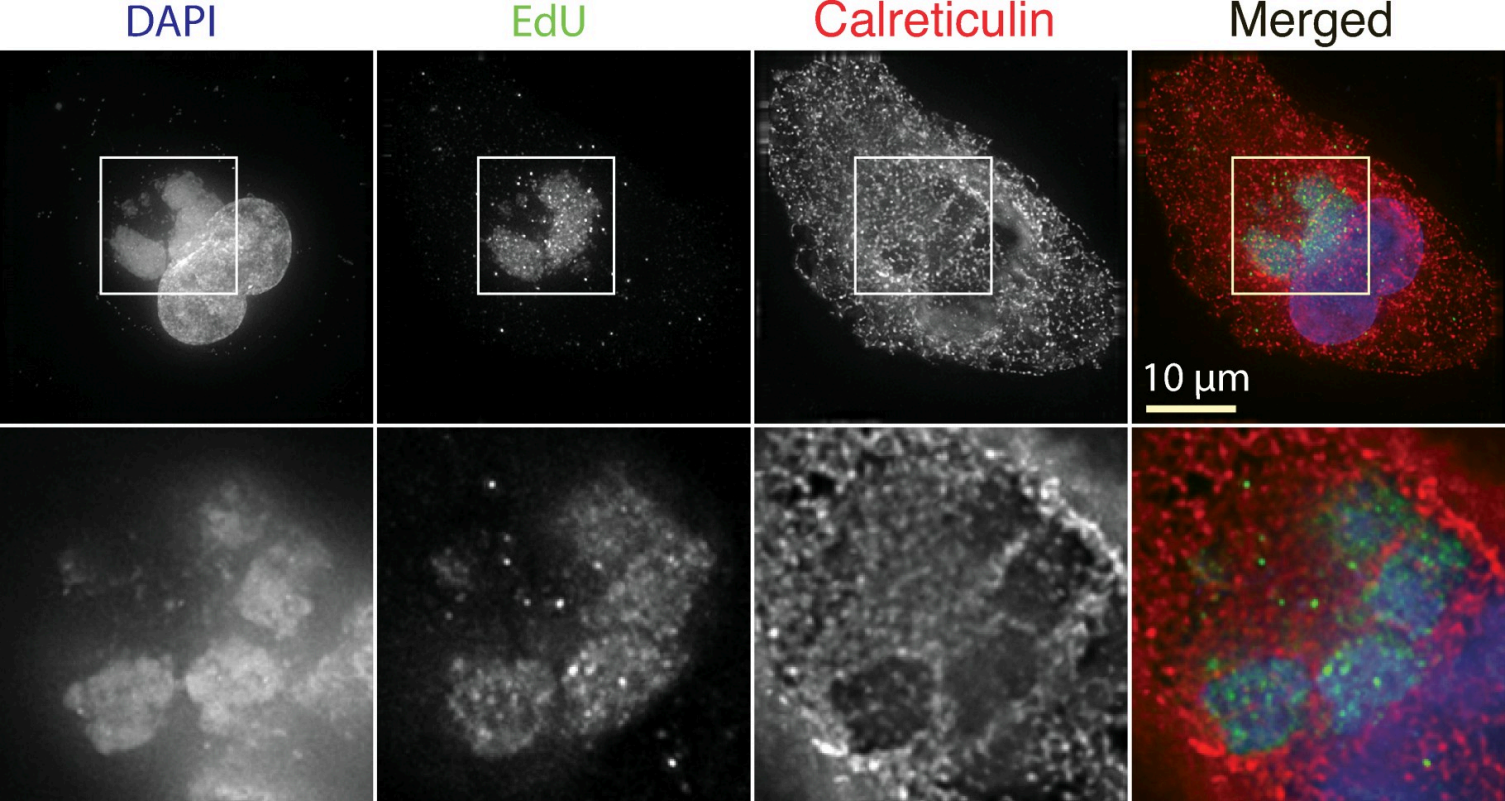
Arrangement of virus factories in a VACV-infected cell. BSC40 cells were infected with VACV strain WR at a MOI = 5 and labelled for 15 min with EdU beginning at 5:45 post-infection. At 6 hr the cells were fixed and processed to detect the EdU label, bulk DNA (DAPI), and the ER marker calreticulin. The top row shows the infected cell at lower magnification and combines all of the z-stacks in a projected image. The bottom row shows a projection of 7x125 nm z-stacks, located in the approximate middle of the cell. In these more highly magnified images one sees calreticulin-labelled channels separating at least four EdU- and DAPI-labelled factories.

VACV factories develop in an environment densely populated with rough ER and this could provide an impediment to factory-factory interactions [8, 27]. To better characterize the spacial relationships between the various cell and virus structures at times just preceding when recombinants start to be detected, we infected BSC40 cells with VACV strain WR, pulse-labelled the cells with EdU for 15 min, and processed the fixed cells at 6 hr post-infection to detect the DNA and EdU as well as the ER marker calreticulin. In this example (Fig 4) several factories are seen at lower magnifications, surrounded by an extensive network of calreticulin-tagged structures. The projection of the whole-cell image from 49 z-stacks tends to obscure the separation between the cytoplasm and the viroplasm in these projected images (Fig 4, upper panels). However, the images assembled from seven 125 nm z-stacks showed clearly that at least four factory elements are separated from each other by calreticulin-rich materials in this region of the cell (Fig 4, bottom). That these are still sites of replication is illustrated by the associated EdU label. Insofar as calreticulin serves as a proxy for ER membranes, this analysis showed that the ER, and other associated cytoplasmic components, creates boundaries that separate regions of viroplasm in each factory.

**Fig 5:**
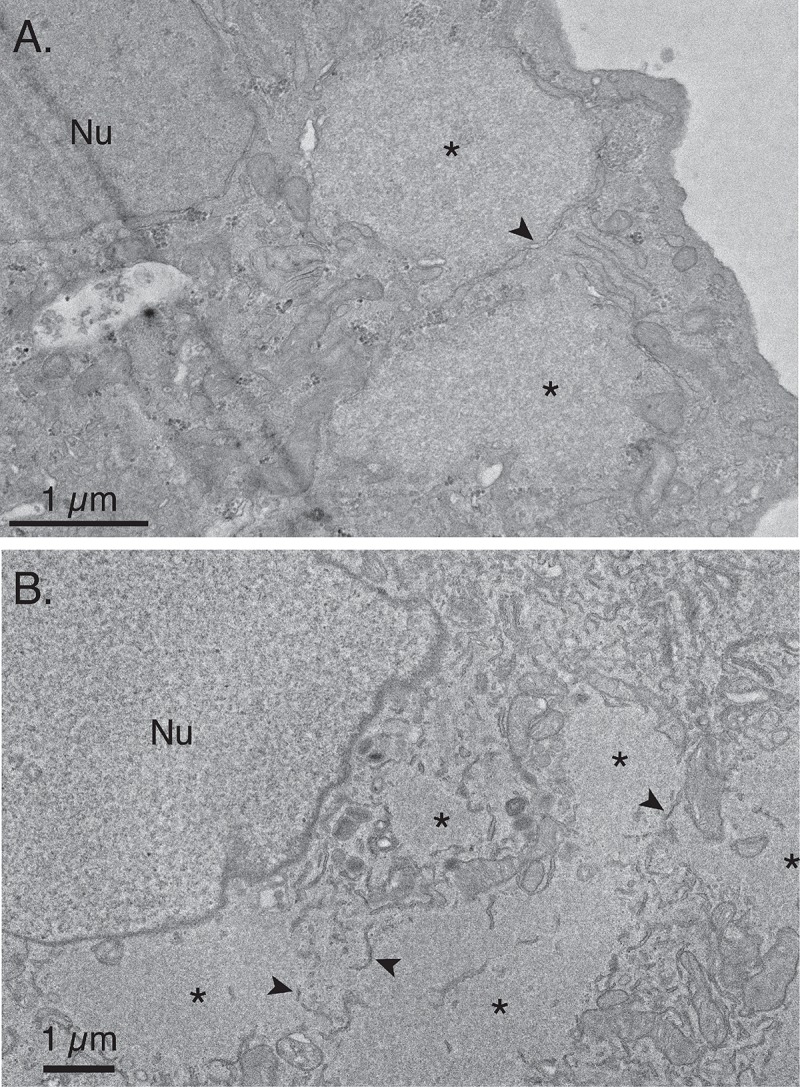
Structures surrounding the edges of virus factories. BSC40 cells were infected with VACV at a MOI = 5 for 3.5 hr (A) or 4 hr (B) and processed for transmission electron microscopy (TEM). At 3.5 hr post-infection the viroplasm (*) presents a uniform pattern of staining and the factories are beginning to lose their once spherical appearance. Membranes, some double layered (arrows), and mitochondria are commonly seen along the edges of these factories. At 4 hr post-infection (B) some factories have migrated to a position adjacent to the nucleus (Nu) and membrane fragments are sometimes also seen surrounding and within the factory.

Calreticulin is a well-established ER marker [33], but the staining seen using fluorescence microscopy (Fig 4) provided no information regarding the integrity of the ER membranes that are presumed to surround the virus factories. Nor can one see what else might occupy these channels. To investigate this question further, we used transmission electron microscopy to image VACV-infected cells (Fig 5). We first studied cells fixed at earlier time points (3.5–4.0 hr post-infection), when the factories are reportedly maximally wrapped in ER membranes [8] and at least an hour before the first IV particles are beginning to be assembled (Fig 1). This timing also precedes the appearance of the first recombinants by several hours. At 3.5 hr post-infection, membranous structures were clearly seen alongside the edges of the virus factories and we also saw a channel of cytoplasm dividing the two factories (Fig 5A). We saw no evidence that there might be any mixing of the two factory’s contents, at least in this section. Another example, captured at 4.0 hr post-infection, illustrates the more amorphous factory structures that begin to appear later in infection (Fig 5B). In this electron micrograph one begins to see what looks like juxtaposed viroplasm in places, with membrane remnants lying in between what might have been the point of contact (Fig 5B). One also sees mitochondria lying along the borders and in-between some factories. These results reinforce the earlier demonstration that each viral factory is surrounded by ER membranes, although the continuity of the wrapping membranes that was reported for very early factories (“mini-nuclei” [8]) is not seen at these later times in factory development. We also see some evidence that remnants of these membranes persist following factory collisions.

### Tracking the development of viral factories with correlative light and electron microscopy (CLEM)

**Fig 6:**
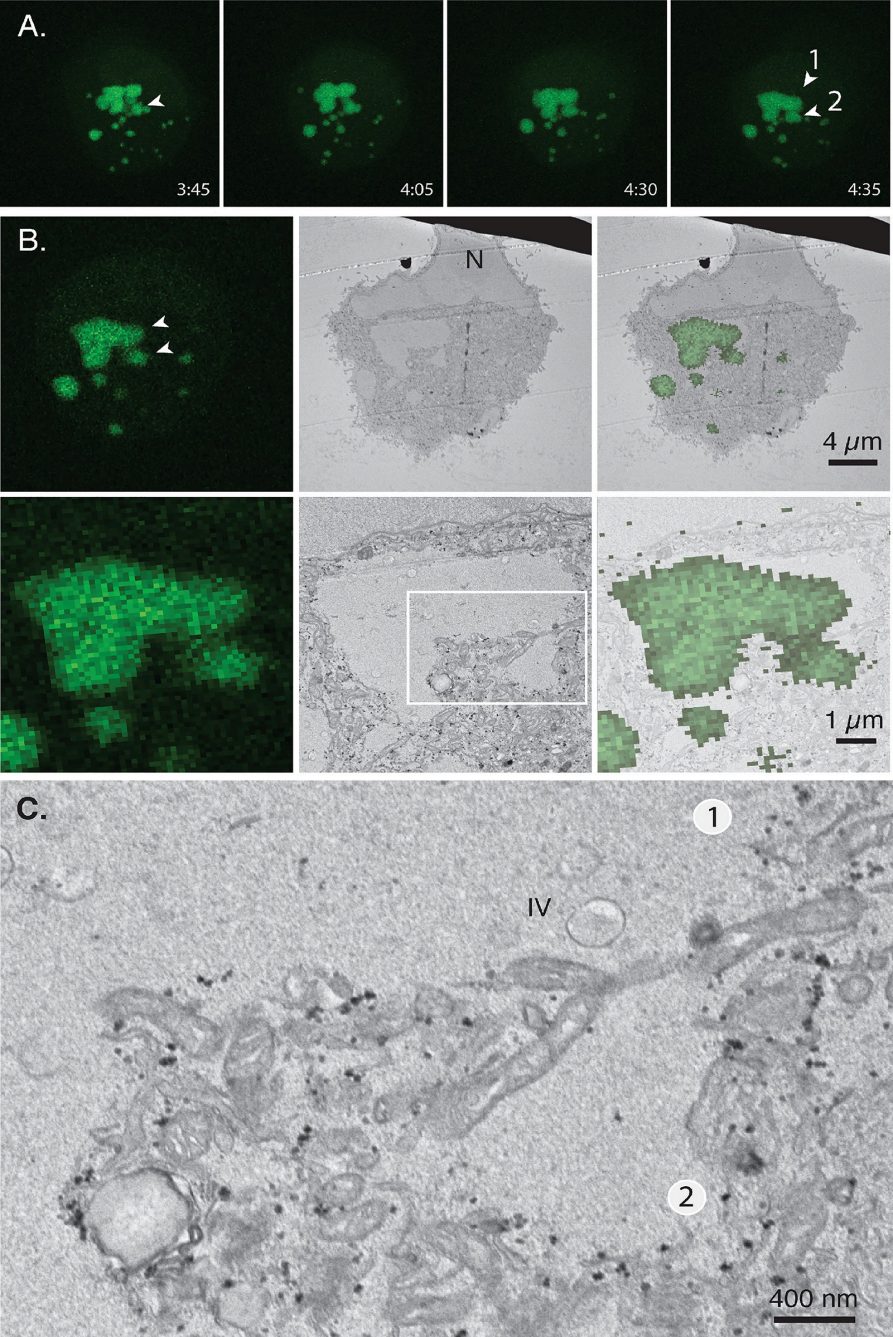
Correlative light and transmission electron microscopy (example 1). (A) BSC40-eGFP-cro cells were infected with VACV at a MOI = 5 and the factory development tracked until a region of collision was identified, between two different factories (“1” and “2”, arrows). A projected image is shown. The cells were fixed and processed for TEM at 4:40 post-infection. (B) Image correlation. The last fluorescence image (left) is shown at the same magnification as an image obtained by TEM (centre). The light and electron micrographs could be well aligned using the different factories as fiduciary markers (right) although it is not possible to perfectly align the images due to slight differences in the optical and TEM image planes. (C) Magnified view of the region surrounding the point of collision between two factories. A variety of cell structures are seen still separating the two factories including membranous debris and many clusters of mitochondria. Also seen in these images are a few crescent structures and IV characteristic of this time point.

The static images seen in Fig 5 provide no insights into the earlier history of the different virus factories. This makes it difficult to interpret the significance of the membrane fragments and other cell contents that separate different regions of viroplasm. To gain some insights into what events preceded the formation of these structures we used a combination of light and electron microscopy to track the development of the factories and then image the observed points of contact. This required that one Correlate the last Light image with micrographs produced using Electron Microscopy, hence the term CLEM. BSC40-eGFP-cro cells were infected with VACV at a MOI = 5 and the developing factories tracked optically until a collision event was observed at about 4:35 post-infection (Fig 6A and S2 Video). The infection was stopped within the next 5 min, and the cell monolayer processed for transmission electron microscopy (TEM). Sections were acquired aligned as closely as possible with the same X-Y plane captured in the optical images. The two images were subsequently aligned, taking advantage of the distinctive patterns formed by the cells and their contents (Fig 6B). Interestingly, one sees a variety of cytoplasmic components trapped between the two recently collided factories (Fig 6C), including some mitochondria. Elsewhere one sees more traces of fragmented cell membranes and there was no obvious sign of mixing of the viroplasm in the short time after the collision event.

**Fig 7:**
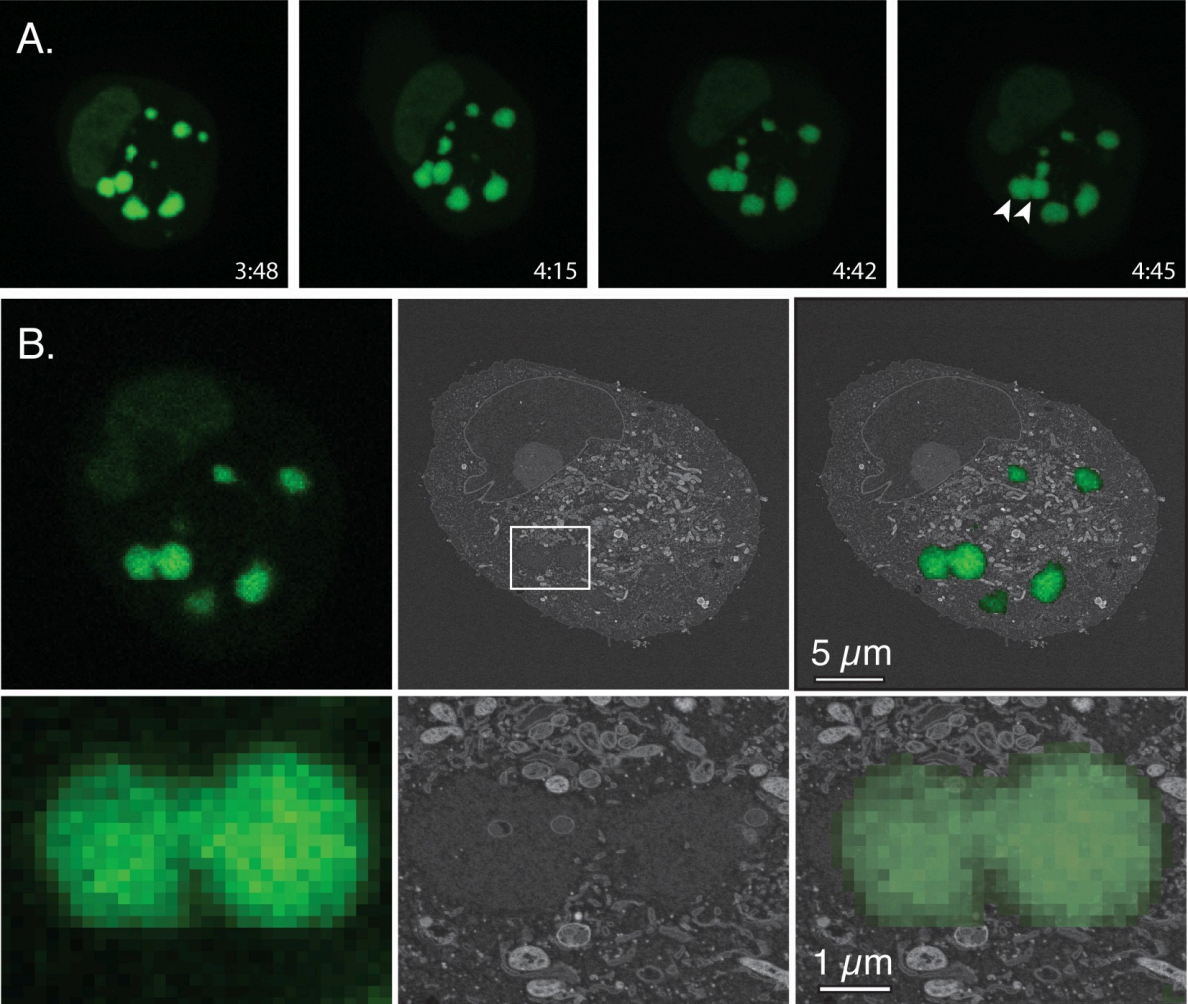
Correlative light and scanning electron microscopy (example 2). BSC40-eGFP-cro cells were infected with VACV at a MOI = 5 and factory development tracked as described. After identifying a site of factory collision, the cells were fixed and processed for SEM. (A) Stills from the live-cell portion of the experiment. Each captures data compiled over 101 z-stacks. A collision between two factories is indicated (arrows) in the image captured at 4:35 post-infection. (B) CLEM analysis of the collision event showing the alignment of the optical and electron images (top row) and an enlargement of the region surrounding the two factories of interest (bottom row). The optical image comes from one z-stack still. By this point one sees little evidence of any contiguous structures separating the viroplasm from the cytoplasm in the SEM image, but remnants of cytoplasmic components are still seen in the space between the two factories. Virus crescents and IV are also beginning to appear.

This experiment was repeated but imaged using scanning rather than transmission electron microscopy (Fig 7 and S3 Video). Hence the reversed contrast. We also endeavoured to match the thickness of the SEM sections (50 nm) with a matching slice from the z-stack of comparable dimensions (~50 nm) in an attempt to more accurately realign the different images. However, the z-resolution of a spinning disk confocal microscope extends beyond 50 nm, which means that the representative stills from the live cell portion of the experiment cannot exactly represent 50 nm sections and this tends to confound the realignment process. In this example the boundaries between the two factories are more difficult to determine, although a disconnected trail of fragmented ER-like double-membrane structures can be seen lying in between the two structures (Fig 7B). As in Fig 6, some IV forms are also beginning to appear embedded within the viroplasm.

### Three-dimensional reconstruction of VACV factories

**Fig 8:**
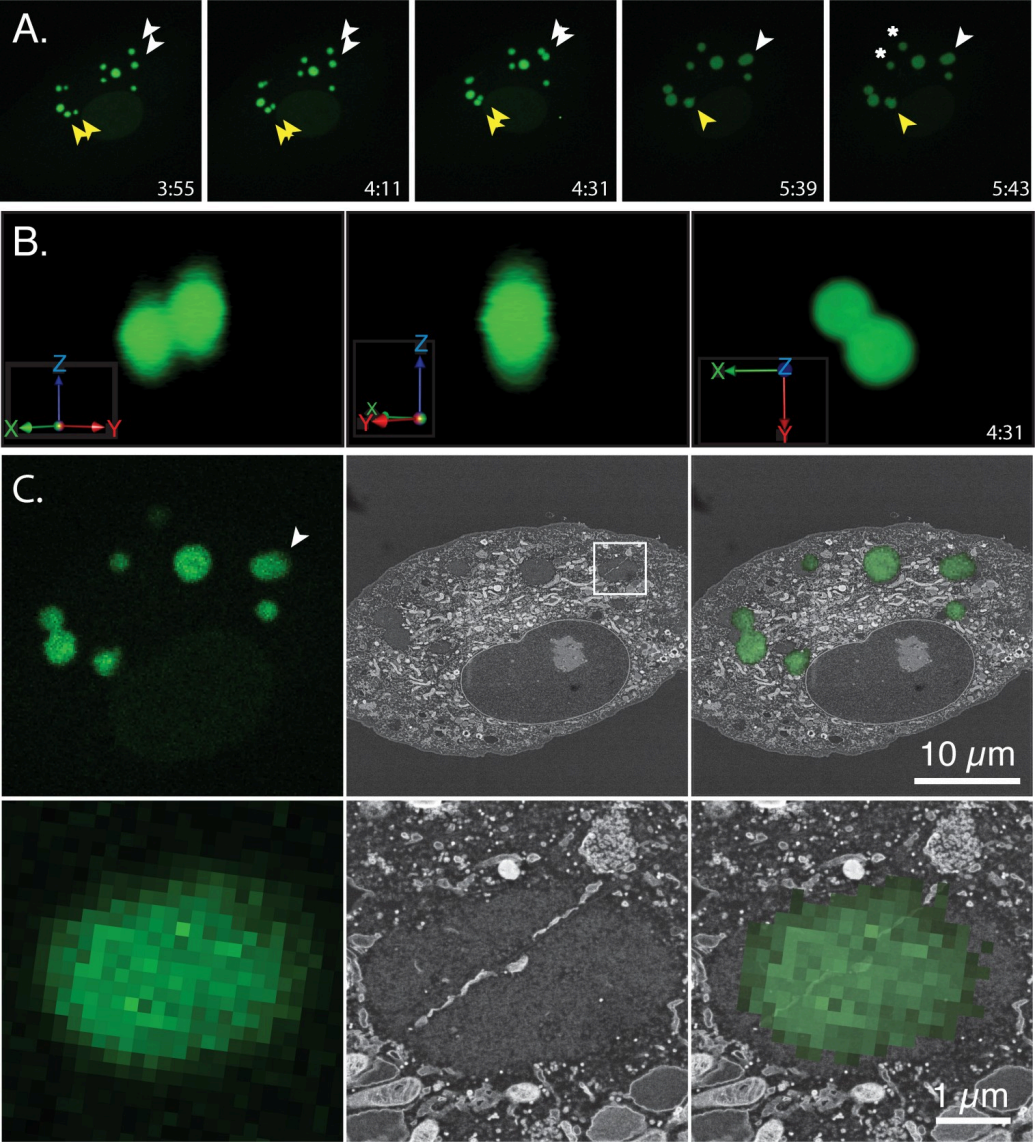
Structure of a fused factory. BSC40-eGFP-cro cells were infected with VACV, tracked, fixed, and processed for SEM as described (Figs 6 and 7). (A) The white arrows mark two factories that collided at 4:31 post infection and then fused to form an ovoid structure that persisted until the reaction was stopped and the cells processed soon after the 5:43 time point. Asterisks highlight two other factories that appeared to have escaped collisions (see text). (B) Three reconstructed 3D volumes showing how the two factories at upper right (white arrow) were oriented relative to each other at the time of collision (4:31). The image showing the XY plane presents the same orientation as the image of these factories in Panel A. (C) CLEM reconstructions showing an alignment of the optical and electron images at two different magnifications. A band of material bisects the factory in this 50 μm thick specimen, along a line roughly tangential to where the two faces collided. The slight misalignment between the optical and electron images in Panel C reflects difficulties perfectly matching the optical and serial sections.

The preceding experiments looked at factories where the co-joined structures still showed evidence of recent collisions between once spherical bodies. To gain some insights into what eventually happens to these structures we used array tomography to build a three-dimensional model of an elliptical factory imaged more than an hour after first contacts. As with the previous experiments, the course of the infection was tracked using live-cell fluorescence microscopy and a well-isolated collision event was noted around 4:31 post-infection (Fig 8A and S4 Video). A 3D-reconstruction of these optical images showed that the two factories occupied slightly different z-planes in the cell at the time of the collision (Fig 8B). This structure was tracked for another 70 min during which it likely rotated through a few degrees counter clockwise, while merging into an oblate spheroid. The cells were then processed for electron microscopy and imaged by SEM. The electron and light images could be generally well correlated using the pattern formed by the eight different factories in this particular cell (Fig 8C, upper panels). An example of one of the 50 nm sections (#16) acquired in this experiment is shown, enlarged in Fig 8C (lower panels) where a band of material can be traced running diagonally though the viroplasm.

**Fig 9:**
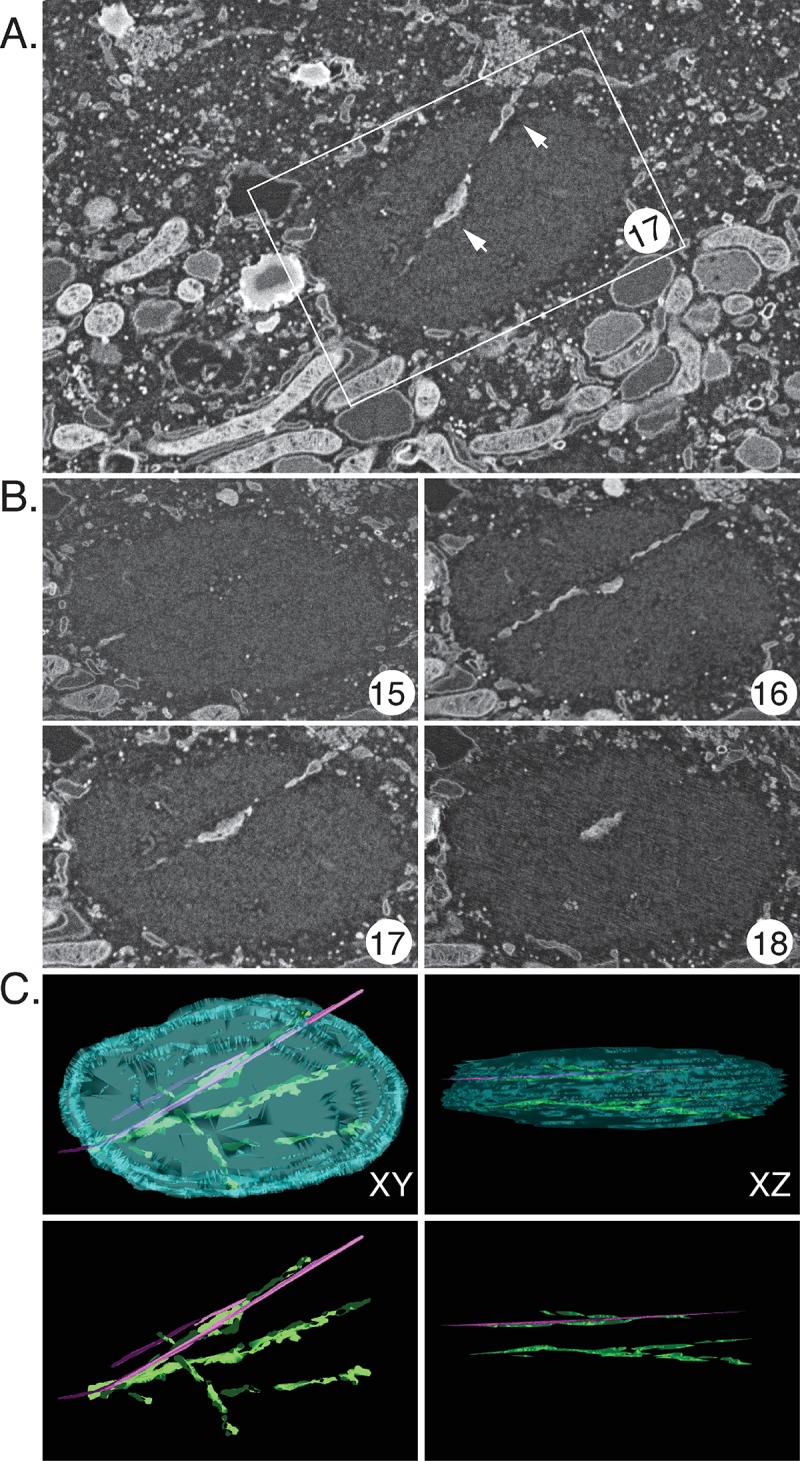
Three-dimensional model of a fused factory assembled from serial sections. (A) SEM image showing the cell matrix surrounding one of the factories. This factory was produced by a single collision event that happened about 80 min before stopping and fixing the cells (Fig 8). This example shows one of 44 serial sections obtained from this cell. The arrows highlight places where doubled membranes, like ER, are visible (B) Four consecutive serial sections encompassing the middle of this factory. The band of material extends only partway from one 50 nm section to the next. Close inspection of section #16 shows that the membranous material tracks along a thin (~25 nm) filament resembling a microtubule. (C) Three-dimensional reconstruction showing the factory boundaries plus the enclosed structures. The boundary between the viroplasm and cytoplasm has been cyan coloured, the bands of infiltrating material are light green, and the 25 nm structures are marked in magenta. A view from the top (XY) and from a longer side (XZ) are displayed with and without the factory boundaries.

**Fig 10:**
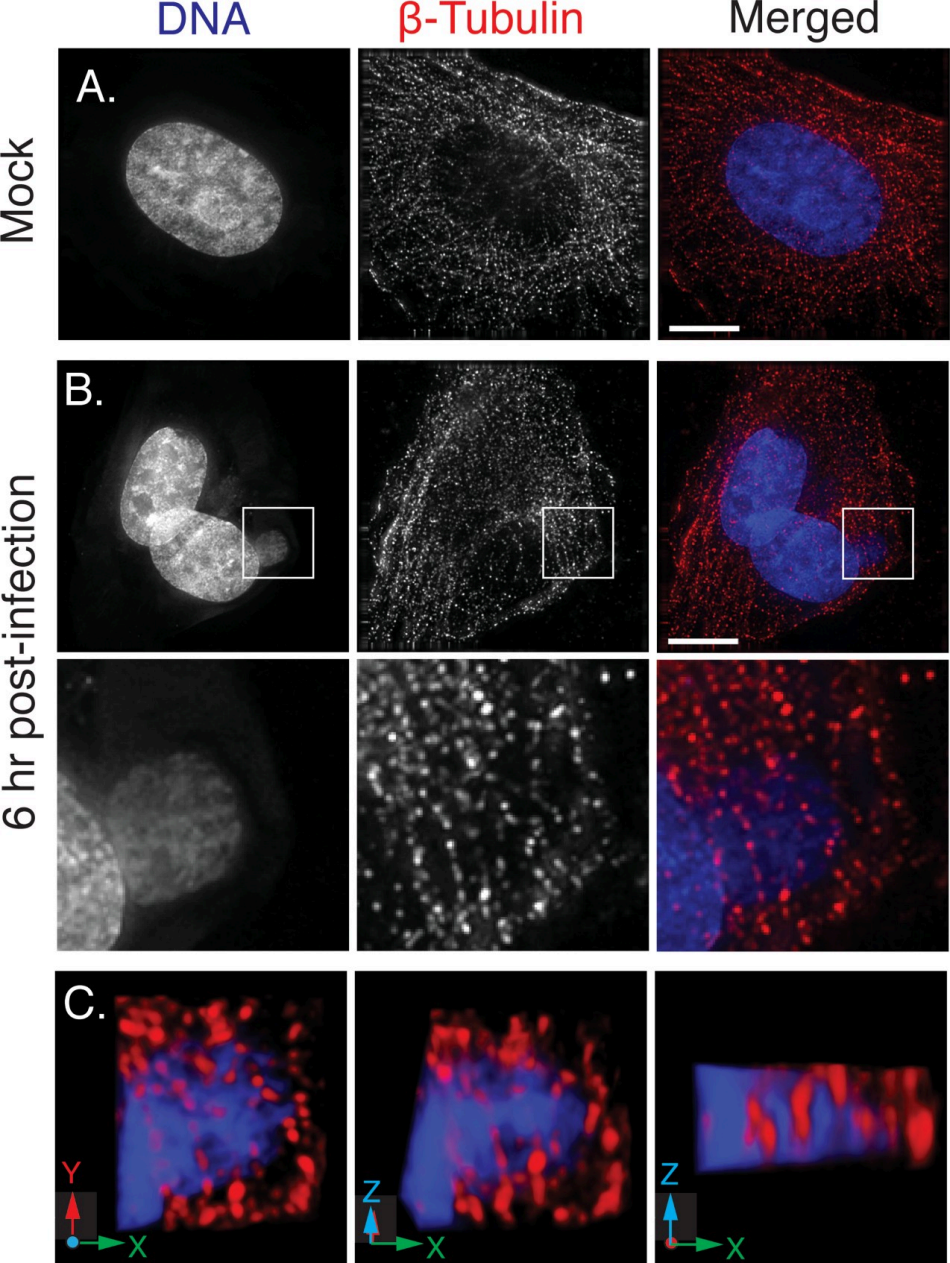
Factories entrain microtubules. BSC40 cells were mock-infected (Panel A) or infected (Panel (B) with VACV at MOI = 1 and then fixed and processed for imaging at 6 hr post-infection. The projections encompass only the image planes spanning the viroplasm. Panel C provides a 3D reconstruction of the region surrounding the virus factory shown in panel B, plus two images produced by consecutive 45˚ rotations of the model around the x-axis. At least two threads of microtubules appear to pass through the body of the factory. To preserve and detect the microtubule structures the cells were fixed at 37˚ in 4% PFA and 0.2% glutaraldehyde, treated with 0.2% sodium borohydride for 10 min and then stained with a mouse anti-tubulin primary antibody plus goat-anti-mouse secondary antibody. Bar = 15 μm.

The serial sections acquired in this experiment were used to trace these banded structures through the remainder of the factory. Fig 9A shows another one of these sections (#17) embedded within the surrounding cytoplasmic matrix. The band of material extends into the cytoplasm and is probably ER judging by the appearance of the paired membranes (arrows). In the adjacent sections one can see that this material does not extend far up or down in the z-plane. It has mostly disappeared by section #18 (Fig 9B). Fig 9C shows a 3D reconstruction of this factory which was assembled by aligning individual micrographs from serial sections extending through the thickness of the factory. This model showed that there were several linear channels traversing the factory, some aligned (as in section #16) along what would have been roughly a tangent to the two colliding faces of the merging factories. These channels also contained several thread-like structures running through the factories, although what is seen may underestimate their abundance as they’d be hard to see in cross-section (Fig 9C, magenta tags and S5 Video). These structures appear to be microtubules judging by their size (~25 nm). The presence of microtubules extending through VACV factories was subsequently confirmed using fluorescence microscopy (Fig 10).

**Fig 11:**
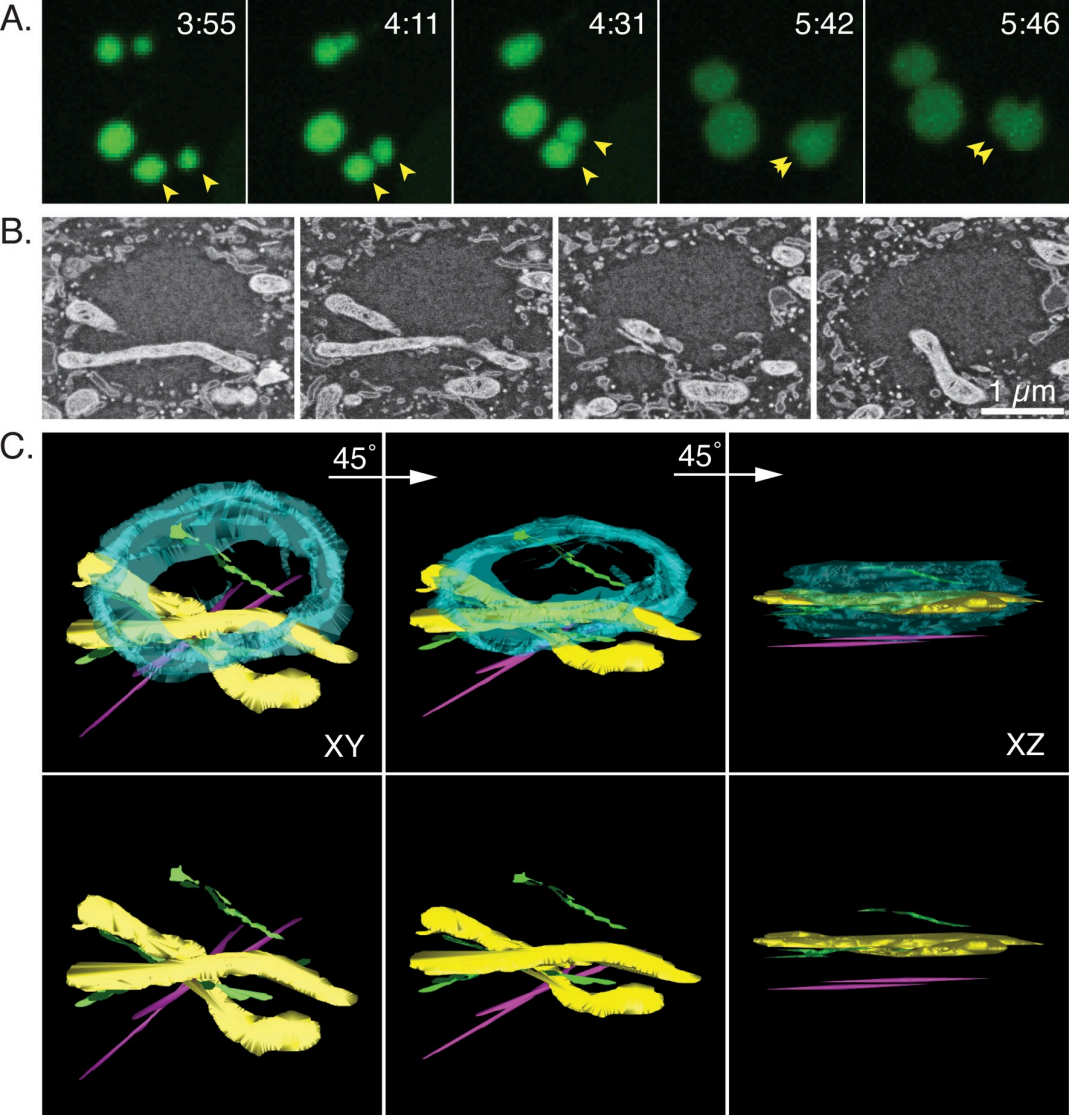
Other entrained structures. (A) A magnified view of the live-cell image in Fig 8A showing a partial collision between two factories (yellow arrows). (B) Four consecutive serial sections through the factory fixed shortly after the 5:46 hr time point. (C) A three-dimensional reconstruction extending through the entirety of the lower factory and up to the boundary with upper one. The boundaries between the viroplasm and cytoplasm have been cyan coloured, the mitochondria yellow, the bands of infiltrating material light green, and the 25 nm structures are marked in magenta. A view from the top (XY) and from a longer side (XZ) are displayed along with a projection along an intermediate rotation axis.

These are not the only cell components that can be trapped in larger virus factories. A second partial collision was seen in these cells (Fig 8A, Fig 11A yellow arrows) and when this event was examined, several sections through this factory clearly contained various cell constituents (Fig 11B). A 3D reconstruction of this structure showed that the lower factory enclosed mitochondria and some ER as well as contacting at least two microtubules (Fig 11C and S6 Video). However, this image is a bit misleading in that the junction with the upper factory (which was only partially captured in the EM sections) likely lies a bit above the plane of the mitochondria, suggesting that the mitochondria could also have been enclosed within the lower factory prior to the partial collision.

These are technically challenging experiments and so it is difficult to produce great numbers of 3D CLEM reconstructions of late merged VACV factories. We did examine the serial sections spanning two of the smaller factories that were probably not fusion products (Fig 8A, asterisks). This hypothesis was based on the fact that these factories weren’t involved in any collisions during the growth and tracking phase, plus any earlier collisions would have tended to create relatively larger structures [28]. A search through these sections did not detect any enclosed material beyond the viroplasm (S1 Fig). Unfortunately, with so few examples, the absence of entrained materials in these smaller factories doesn’t offer solid proof that the structures seen in larger factories are a product of collisions. They could still just have been occluded as the factories grew. However, what is apparent is that once two or more factories have overcome any initial obstacles to fusion and finally collided and/or merged, the disconnected nature of any remaining entrapped materials doesn’t seem sufficient to offer intractable obstacles to mixing and recombination of the virus DNA.

## Conclusions

During the early stages of infection, each poxvirus factory comprises a replicating clone of virus DNA and recombinants can’t be formed until different DNAs are mixed in an environment still proficient at replication and recombination. Such constraints on interviral recombination aren’t restricted to cytoplasmic poxviruses, as they are also observed in cell nuclei where co-infecting herpes viruses have been shown to form analogous clonal replication compartments [34]. In our case these systems are most active 3–4 hr post infection in poxvirus-infected cells, but DNA polymerase/recombinase activity [21, 35] is still detectable 5–6 hr post infection (Fig 2) when the first virus-by-virus recombinants are detected (Fig 3). We had initially hypothesized that the ER membranes that closely wrap factories at early stages of infection [8] might serve as a substantial barrier to mixing and recombination. However, as the factories grow the continuity of these structures is lost (e.g. Fig 5), perhaps due to the scavenging of the membranes during virus assembly or simply through expansion of the viroplasm. Consequently, it is difficult to imagine that these structures would offer much of an impediment to mixing except perhaps at the earliest stage of infection. What one sees is that a lot of cytoplasmic constituents seem to become trapped, if only transiently, at the interfaces between colliding factories. The most identifiable structures are mitochondria and membrane fragments (Figs 6 and 7) and judging by how long different factories can sometimes “dance” around each other before fusing, these cytoplasmic constituents likely offer a significant barrier to fusion.

Of course, factories eventually fuse to form structures that in an optical image present an ovoid appearance (Fig 8). CLEM is a technically challenging process that provides insights into the internal structures of these and other colliding factories albeit for limited numbers of cells (Figs 8, 9, and 11). Interestingly the larger collision and fusion products still entrain some identifiable cellular materials like ER and mitochondria (Figs 9 and 11). They also enclose structures resembling ER-wrapped microtubules although whether these materials were trapped during the collision process or are simply adventitious intrusions is difficult to state conclusively. The ER does assemble along paths established by a microtubular network [36] and thus such structures could have been acquired by a process independent of collisions. The presence of factory-associated microtubules (Fig 10) is interesting as it would offer a path for MV exit [37] (like a “fireman’s pole”) and also account for the reports of nocodazole-resistant microtubule fragments in VACV-infected cells [38]. It could also create an anchor that would also constrain further factory movement and fusion.

What these observations also show is that once factories have collided and formed a tight association, there doesn’t seem to be any obvious remaining barriers to mixing the viroplasma (Fig 9). Yet one sees that something still constrains DNA [28] and protein [9] mixing between factories. The simplest additional explanation would be that the viroplasm is so dense (or viscous) that this can further impede DNA mixing even in fused factories. It is striking that one can readily identify a distinct boundary between viroplasm and cytoplasm throughout most of the infection, despite there being no obvious barrier that might impede the diffusion of the viroplasm beyond the earliest stage of infection. What might finally disrupt this barrier to DNA mixing is packaging, and it may not be a coincidence that virus-by-virus recombination is typically first detected at 5–6 hr post-infection (Fig 3), by which time the assembly of large numbers of IV and MV (~10% of the 24 hr yield, Fig 1) may have begun to upset the stability of the viroplasm. Of course, by this point the replication machinery is well into decline, providing only a narrow window of opportunity for assembling recombinants before this machinery disappears and the genomes are isolated by packaging.

## Materials and methods

### Viruses and cells

BSC40-eGFP-cro cells were constructed as described [28] and grown on modified Eagle’s medium (MEM) or FluoroBrite^TM^ Dulbecco’s modified Eagle’s medium (DMEM). Both were supplemented with sodium pyruvate, antibiotics/antimycotics, L-glutamine, non-essential amino acids, and 5% FetalGro^®^ (RMBIO). The cells were seeded on glass cover slips for fixed-cell experiments or glass-bottomed and gridded 35mm dishes for live-cell studies and CLEM. The construction of two recombinant derivatives of VACV strain WR [VACV-pE/L-mCherry(t) and VACV-pmCherry-cro are described in Paszkowski et al. [27]. VACV-A5-YFP was a kind gift of Dr. B. Moss [9]. The cells were infected with virus at 4°C in serum-free media supplemented with 10 mM HEPES buffer. After 1 hr the cells were washed twice with cold phosphate buffered saline (PBS), pre-warmed MEM or Fluorobrite DMEM (for live-cell imaging) was added, and the culturing continued at 37˚C until imaged and fixed.

### Fluorescence microscopy

To process the cells for immunofluorescence microscopy they were washed once with PBS and fixed with 4% fresh paraformaldehyde for ≥30 minutes at 4°C. The cells were incubated for 20 minutes at room temperature in PBS containing 0.1% Triton X-100 (PBS-T) plus 0.1M glycine, washed three times with PBS-T, and blocked with 3% BSA in PBS-T for 30 min. The cells were then incubated with the primary antibody for 90 min at room temperature (or at 4°C overnight), washed three times with PBS-T, and incubated with a secondary antibody for 45 minutes at room temperature. The cells were mounted in ProLong^™^ Gold Antifade (ThermoFisher) and sealed with nail polish. To image live cells the dish was sealed with Parafilm and transferred to a 37˚C microscope stage ~1 hr before beginning image capture. FITC and RFP filter sets were used to detect enhanced green and mCherry fluorescent proteins, respectively. The cells were subsequently fixed with 4% paraformaldehyde for later optical imaging or with 2% glutaraldehyde for EM. For confocal microscopy, samples were imaged using an Olympus IX-81 spinning disc confocal microscope (40x/NA = 1.3). Alternatively, samples were imaged using a DeltaVision OMX structured illumination microscope (60x/NA = 1.42) for super-resolution microscopy. Details are found in Kieser et al. [39].

### EdU labelling

A 20 μM solution of 5-ethynyl-2’-deoxyuridine (EdU) in warmed media was added to an equal volume of cells in culture media at the indicated times. After 15 min the EdU was removed and the cells washed once with warm PBS before either adding fresh media or proceeding to the fixation step. After fixing and blocking the cells with 3% BSA in PBS-T, click chemistry was used to couple an AlexaFluor^®^ azide moiety onto the incorporated EdU [39]. The cells were washed three times with PBS-T and processed for fluorescence microscopy as above.

### Electron microscopy

To process the cells for TEM, they were fixed in 2% glutaraldehyde in 0.1M cacodylate buffer (CB) for 1 hr at 4°C and then at 20˚C for 1 hr. The cells were washed with CB, post-fixed with 1% OsO_4_ and 1.5% K_4_Fe(CN)_6_ in CB, washed with 0.1M sodium acetate (pH 5.2) and stained with 1% uranyl acetate in sodium acetate buffer in the dark. The samples were dehydrated through a 10–100% ethanol series, embedded in Spurr’s resin, and sectioned using an EM UC7 ultramicrotome. The sections were transferred to carbon-coated grids, stained with 2% uranyl acetate and 2% lead citrate before imaging with a Hitachi H-7650 TEM.

To process the cells for SEM they were first fixed in 2.5% glutaraldehyde in CB for 30 min at 20˚. The cells were post-fixed with 1% OsO_4_ and 1% K_4_Fe(CN)_6_ in CB and treated with 1% thiocarbohydrazide before staining with 1% OsO_4_ followed by 1% uranyl acetate and lead aspartate, and dehydrated using a graded ethanol series. The cells were embedded in Durcupan resin before collecting serial sections. A Hitachi S-4800 field emission gun SEM was used to image the sections.

### Image analysis

Volocity software was used for particle counts. Each image was split into three replicate images: one marker image showing D13+A5, a second marker image showing B5+A5, and a third image showing only A5. “Regions of interest” (ROI) were used to identify the different virus forms on the marker images (e.g. ROI’s were used to tag IV in the D13/A5 image) and then the ROI’s were transferred to the A5 image for counting. The A5 image was also used to count total virus. The ROI’s mapped in a single marker image could be used to count either IV or IEV, but not both. Therefore, we had to generate separate ROI’s for each of the marker images to count both IV and IEV. The number of MV were calculated by subtracting the number of IV and IEV from the total count. The data were collected and pooled from ten cells per time point and combined three separate experiments.

FIJI [40] and IMOD [41] were used to produce the correlative images. An image sequence was first created using serial EM images and these images were registered using FIJI’s TrakEM2 plug-in. The realigned images were then transferred to IMOD to generate the 3D model. Separate contours were generated for each area of interest. In the examples shown here, contours were created that highlight a selection of materials found near and inside viral factories along with another contour that marked the viroplasm/cytoplasm boundary. These contours were used by IMOD to generate the 3D-models.

## Supporting information

S1 Fig Serial sections through a smaller factory that does not appear to have been involved in a collision or fusion event. The micrographs show every third of the 30 sections that spanned the larger of the two factories marked with an asterisk in Fig 8A.
(TIF)

S1 Table Data supporting Figs 1D and 2B. (XLSX)

S1 Video Factory dynamics (collisions) and recombinant formation. The video shows factory movement in the 4 hr leading up to and after EdU was added at the 6 hr time point. BSC40-eGFP-cro cells were co-infected with VACV-pE/L-mCherry(t) and VACV-pmCherry-cro viruses at a MOI = 2.5 for each virus. Recombination permits expression of mCherry-cro protein, which is first detectable at the 5:20 hr time point. The reaction was stopped and the cells processed to detect the incorporated EdU at the 6:35 hr time point (see Fig 3).
(MP4)

S2 Video Factory dynamics (collisions). The video shows factory movement in the 50 min leading up to when the reaction was stopped and the infected cells were processed for transmission electron microscopy. The beginning of the video was at 3:45 hr post-infection (see also Fig 6).
(MP4)

S3 Video Factory dynamics (collisions). The video shows factory movement in the 60 min leading up to when the reaction was stopped and the infected cells were processed for scanning electron microscopy. The beginning of the video was at 3:48 hr post-infection (see also Fig 7).
(MP4)

S4 Video Factory dynamics (fusion). The video shows factory movement in the 110 min leading up to when the reaction was stopped and the infected cells processed for scanning electron microscopy. The beginning of the video was at 3:55 hr post-infection (see also Fig 8).
(MP4)

S5 Video Reconstructed model of a factory formed by fusion of two separate factories. IMOD [41] was used to create the model from 44 serial sections through the centre of the infected cell. The boundaries between the viroplasm and cytoplasm have been cyan coloured, the bands of infiltrating material are light green, and the 25 nm microtubular structures are marked in magenta (see also Fig 9).
(MP4)

S6 Video Reconstructed model of a factory associated with the collision and partial fusion of two separate factories. The model extends through the entirety of the lower factory (33 serial sections) and up to the boundary with the upper one (see also Fig 11). The boundaries between the viroplasm and cytoplasm have been cyan coloured, the mitochondria yellow, the bands of infiltrating material light green, and the 25 nm structures are marked in magenta.
(MP4)

## References

[1] Yang Z, Cao S, Martens CA, Porcella SF, Xie Z, Ma M, et al Deciphering poxvirus gene expression by RNA sequencing and ribosome profiling. J Virol. 2015;89(13):6874–86. 10.1128/JVI.00528-15 25903347 PMC4468498

[2] Yang Z, Reynolds SE, Martens CA, Bruno DP, Porcella SF, Moss B. Expression profiling of the intermediate and late stages of poxvirus replication. J Virol. 2011;85(19):9899–908. 10.1128/JVI.05446-11 21795349 PMC3196450

[3] Teale A, Campbell S, Van Buuren N, Magee WC, Watmough K, Couturier B, et al Orthopoxviruses require a functional ubiquitin-proteasome system for productive replication. J Virol. 2009;83(5):2099–108. 10.1128/JVI.01753-08 19109393 PMC2643736

[4] Mercer J, Snijder B, Sacher R, Burkard C, Bleck CK, Stahlberg H, et al RNAi screening reveals proteasome- and Cullin3-dependent stages in vaccinia virus infection. Cell Rep. 2012;2(4):1036–47. 10.1016/j.celrep.2012.09.003 23084750

[5] Liu B, Panda D, Mendez-Rios JD, Ganesan S, Wyatt LS, Moss B. Identification of Poxvirus Genome Uncoating and DNA Replication Factors with Mutually Redundant Roles. J Virol. 2018;92(7). 10.1128/JVI.02152-17 PMC5972866 29343579

[6] Cairns J. The initiation of vaccinia infection. Virology. 1960;11:603–23. 10.1016/0042-6822(60)90103-3 13806834

[7] Dales S, Siminovitch L. The development of vaccinia virus in Earle's L strain cells as examined by electron microscopy. J Biophys Biochem Cytol. 1961;10:475–503. 10.1083/jcb.10.4.475 13719413 PMC2225098

[8] Tolonen N, Doglio L, Schleich S, Krijnse Locker J. Vaccinia virus DNA replication occurs in endoplasmic reticulum-enclosed cytoplasmic mini-nuclei. Mol Biol Cell. 2001;12(7):2031–46. 10.1091/mbc.12.7.2031 11452001 PMC55651

[9] Katsafanas GC, Moss B. Colocalization of transcription and translation within cytoplasmic poxvirus factories coordinates viral expression and subjugates host functions. Cell Host Microbe. 2007;2(4):221–8. 10.1016/j.chom.2007.08.005 18005740 PMC2084088

[10] Weisberg AS, Maruri-Avidal L, Bisht H, Hansen BT, Schwartz CL, Fischer ER, et al Enigmatic origin of the poxvirus membrane from the endoplasmic reticulum shown by 3D imaging of vaccinia virus assembly mutants. Proc Natl Acad Sci U S A. 2017;114(51):E11001–E9. 10.1073/pnas.1716255114 29203656 PMC5754806

[11] Harrison K, Haga IR, Pechenick Jowers T, Jasim S, Cintrat JC, Gillet D, et al Vaccinia Virus Uses Retromer-Independent Cellular Retrograde Transport Pathways To Facilitate the Wrapping of Intracellular Mature Virions during Virus Morphogenesis. J Virol. 2016;90(22):10120–32. 10.1128/JVI.01464-16 27581988 PMC5105650

[12] Czarnecki MW, Traktman P. The vaccinia virus DNA polymerase and its processivity factor. Virus Res. 2017;234:193–206. 10.1016/j.virusres.2017.01.027 28159613 PMC5476500

[13] Rochester SC, Traktman P. Characterization of the single-stranded DNA binding protein encoded by the vaccinia virus I3 gene. J Virol. 1998;72(4):2917–26. 9525612 10.1128/jvi.72.4.2917-2926.1998 PMC109737

[14] Welsch S, Doglio L, Schleich S, Krijnse Locker J. The vaccinia virus I3L gene product is localized to a complex endoplasmic reticulum-associated structure that contains the viral parental DNA. J Virol. 2003;77(10):6014–28. 10.1128/JVI.77.10.6014-6028.2003 12719593 PMC154049

[15] Magee WC, Shahhosseini S, Lin YC, Suresh MR, Evans DH. Production and characterization of antibodies against vaccinia virus DNA polymerase. J Virol Methods. 2009;161(1):44–51. 10.1016/j.jviromet.2009.05.012 19477201

[16] DeLange AM, McFadden G. Sequence-nonspecific replication of transfected plasmid DNA in poxvirus- infected cells. Proc Natl Acad Sci U S A. 1986;83(3):614–8. 10.1073/pnas.83.3.614 3003742 PMC322914

[17] De Silva FS, Moss B. Origin-independent plasmid replication occurs in vaccinia virus cytoplasmic factories and requires all five known poxvirus replication factors. Virol J. 2005;2:23 10.1186/1743-422X-2-23 15784143 PMC1079961

[18] Parks RJ, Evans DH. Effect of marker distance and orientation on recombinant formation in poxvirus-infected cells. J Virol. 1991;65(3):1263–72. 1847453 10.1128/jvi.65.3.1263-1272.1991 PMC239901

[19] Evans DH, Stuart D, McFadden G. High levels of genetic recombination among cotransfected plasmid DNAs in poxvirus-infected mammalian cells. J Virol. 1988;62(2):367–75. 2826801 10.1128/jvi.62.2.367-375.1988 PMC250545

[20] Willer DO, Yao XD, Mann MJ, Evans DH. In vitro concatemer formation catalyzed by vaccinia virus DNA polymerase. Virology. 2000;278(2):562–9. 10.1006/viro.2000.0686 11118378

[21] Gammon DB, Evans DH. The 3'-to-5' exonuclease activity of vaccinia virus DNA polymerase is essential and plays a role in promoting virus genetic recombination. J Virol. 2009;83(9):4236–50. 10.1128/JVI.02255-08 19224992 PMC2668504

[22] Yao XD, Evans DH. Characterization of the recombinant joints formed by single-strand annealing reactions in vaccinia virus-infected cells. Virology. 2003;308(1):147–56. 10.1016/s0042-6822(02)00089-2 12706098

[23] Wright WD, Shah SS, Heyer WD. Homologous recombination and the repair of DNA double-strand breaks. J Biol Chem. 2018;293(27):10524–35. 10.1074/jbc.TM118.000372 29599286 PMC6036207

[24] Esposito JJ, Sammons SA, Frace AM, Osborne JD, Olsen-Rasmussen M, Zhang M, et al Genome sequence diversity and clues to the evolution of variola (smallpox) virus. Science. 2006;313(5788):807–12. 10.1126/science.1125134 16873609

[25] Elde NC, Child SJ, Eickbush MT, Kitzman JO, Rogers KS, Shendure J, et al Poxviruses deploy genomic accordions to adapt rapidly against host antiviral defenses. Cell. 2012;150(4):831–41. 10.1016/j.cell.2012.05.049 22901812 PMC3499626

[26] Qin L, Evans DH. Genome scale patterns of recombination between coinfecting vaccinia viruses. J Virol. 2014;88(10):5277–86. 10.1128/JVI.00022-14 24574414 PMC4019122

[27] Paszkowski P, Noyce RS, Evans DH. Live-Cell Imaging of Vaccinia Virus Recombination. PLoS Pathog. 2016;12(8):e1005824 10.1371/journal.ppat.1005824 27525721 PMC4985154

[28] Lin YC, Evans DH. Vaccinia virus particles mix inefficiently, and in a way that would restrict viral recombination, in coinfected cells. J Virol. 2010;84(5):2432–43. 10.1128/JVI.01998-09 20032178 PMC2820930

[29] Moss B. Poxvirus membrane biogenesis. Virology. 2015;479–480:619–26. 10.1016/j.virol.2015.02.003 PMC4424062 25728299

[30] Roberts KL, Smith GL. Vaccinia virus morphogenesis and dissemination. Trends Microbiol. 2008;16(10):472–9. 10.1016/j.tim.2008.07.009 18789694

[31] Cepeda V, Esteban M. Novel insights on the progression of intermediate viral forms in the morphogenesis of vaccinia virus. Virus Res. 2014;183:23–9. 10.1016/j.virusres.2014.01.016 24468494

[32] McDonald WF, Crozel-Goudot V, Traktman P. Transient expression of the vaccinia virus DNA polymerase is an intrinsic feature of the early phase of infection and is unlinked to DNA replication and late gene expression. J Virol. 1992;66(1):534–47. 1727498 10.1128/jvi.66.1.534-547.1992 PMC238314

[33] Fliegel L, Burns K, MacLennan DH, Reithmeier RA, Michalak M. Molecular cloning of the high affinity calcium-binding protein (calreticulin) of skeletal muscle sarcoplasmic reticulum. J Biol Chem. 1989;264(36):21522–8. 2600080

[34] Kobiler O, Weitzman MD. Herpes simplex virus replication compartments: From naked release to recombining together. PLoS Pathog. 2019;15(6):e1007714 10.1371/journal.ppat.1007714 31158262 PMC6546242

[35] Willer DO, Mann MJ, Zhang W, Evans DH. Vaccinia virus DNA polymerase promotes DNA pairing and strand-transfer reactions. Virology. 1999;257(2):511–23. 10.1006/viro.1999.9705 10329561

[36] Lee C, Ferguson M, Chen LB. Construction of the endoplasmic reticulum. J Cell Biol. 1989;109(5):2045–55. 10.1083/jcb.109.5.2045 2478561 PMC2115887

[37] Hollinshead M, Rodger G, Van Eijl H, Law M, Hollinshead R, Vaux DJ, et al Vaccinia virus utilizes microtubules for movement to the cell surface. J Cell Biol. 2001;154(2):389–402. 10.1083/jcb.200104124 11470826 PMC2150758

[38] Schepis A, Schramm B, de Haan CA, Locker JK. Vaccinia virus-induced microtubule-dependent cellular rearrangements. Traffic. 2006;7(3):308–23. 10.1111/j.1600-0854.2005.00381.x 16497225

[39] Kieser Q, Paszkowski P, Lin J, Evans D, Noyce R. Visualizing Poxvirus Replication and Recombination Using Live-Cell Imaging. Methods Mol Biol. 2019;2023:221–35. 10.1007/978-1-4939-9593-6_14 31240681

[40] Schindelin J, Arganda-Carreras I, Frise E, Kaynig V, Longair M, Pietzsch T, et al Fiji: an open-source platform for biological-image analysis. Nat Methods. 2012;9(7):676–82. 10.1038/nmeth.2019 22743772 PMC3855844

[41] Kremer JR, Mastronarde DN, McIntosh JR. Computer visualization of three-dimensional image data using IMOD. J Struct Biol. 1996;116(1):71–6. 10.1006/jsbi.1996.0013 8742726

