# Supplementary figures and images for: Cytoplasmic factories, virus assembly, and DNA replication kinetics collectively constrain the formation of poxvirus recombinants
Source: PLoS One. 2020 Jan 16;15(1):e0228028. doi: 10.1371/journal.pone.0228028 (PMC6964908; doi:10.1371/journal.pone.0228028)

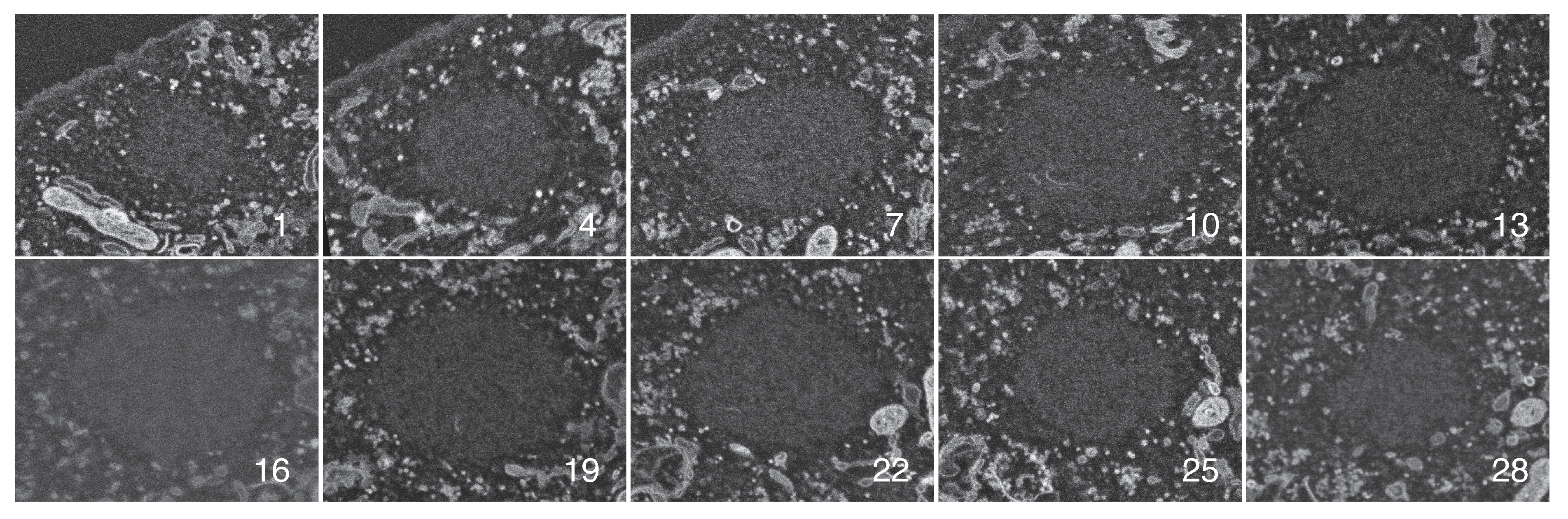

Supplement: S1 Fig — The micrographs show every third of the 30 sections that spanned the larger of the two factories marked with an asterisk in Fig 8A. (TIF) [file pone.0228028.s001.tif]
